# Whole genome sequencing of Aoluguya reindeer (*Rangifer tarandus*) in China

**DOI:** 10.3389/fgene.2023.1243795

**Published:** 2023-08-02

**Authors:** Lulu Shi, Zheng Shi, Mingyue Hu, Mao Wu, Xinjiao Quan, Lihong Qin, Zhongli Zhao, Hao Sun, Laiming Tian, Shouqing Yan

**Affiliations:** ^1^ College of Animal Science, Jilin University, Changchun, China; ^2^ Jilin Province Animal Husbandry and Veterinary Academy of Sciences, Changchun, China; ^3^ Institute of Animal Husbandry and Veterinary, Jilin Academy of Agricultural Sciences, Gongzhuling, China

**Keywords:** Aoluguya reindeer, whole genome sequencing, SNP, genetic relationship, genetic resource

## Introduction

Reindeer (*Rangifer tarandus*) belonging to Artiodactyla order and Cervidae family has a broad range in the Arctic and sub-Arctic regions mainly including Norway, Finland, Sweden, Russia, the United States, and Canada (Ju et al., 2019). Hunting and herding reindeer are of utmost importance to the people residing in the Arctic (Kvie et al., 2016). For centuries, reindeer has been a crucial source of subsistence items for Arctic residents, such as food and fur, and has also played a key role in transportation (Kofinas et al., 2000). Additionally, reindeer has played a pivotal role in the origin and development of numerous indigenous cultures in the North (Baskin, 2000). By studying reindeer, researchers can gain insights into the history of many Arctic cultures. Although the reindeer was added to the Red List of Threatening Species of the International Union for Conservation of Nature (IUCN) in 2015 and were estimated as vulnerable according to the criteria of A2a (http://www.iucnredlist.org/), fortunately it has beed monitored so that we can improve its position.

**FIGURE 1:**
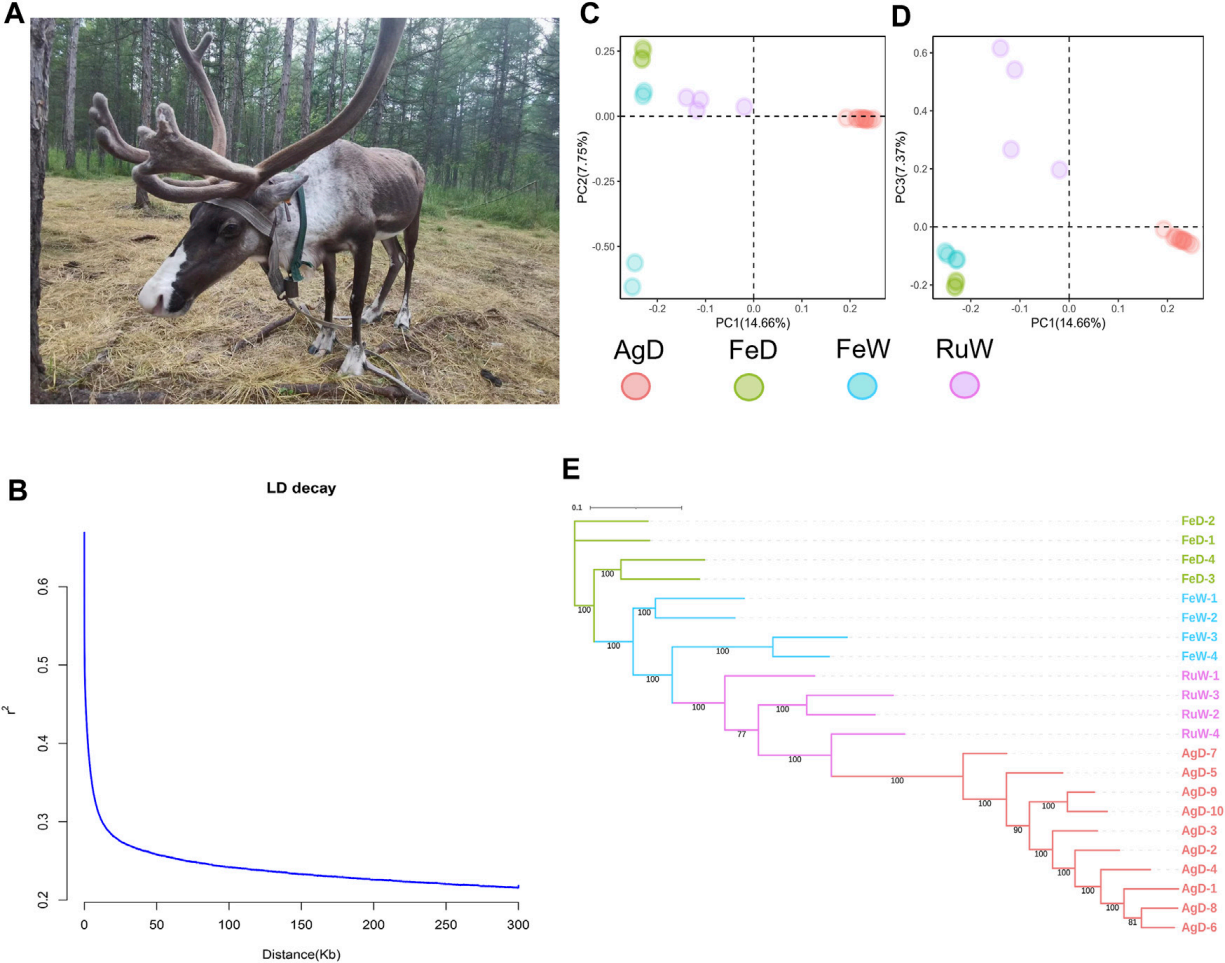
**(A)** The Aoluguya reindeer in China. **(B)** LD decay pattern for AgD at distances up to 300 kb. **(C)** and **(D)** PCA results in four populations. **(E)** The maximum likelihood (ML) tree constructed using RAxML with 100 bootstraps. AgD, Aoluguya reindeer; FeD, Fennoscandian domestic reindeer; FeW, Fennoscandian wild reindeer; RuW, Russian wild reindeer.

Aoluguya reindeer (Figure 1A), located in the Greater Khingan Mountain region of Inner Mongolia in China (50°20′-52°30′N, 120°12′-122°55′E) and domesticated by the Ewenki people, is a population that is currently being bred in the southernmost part of this species habitat and is a valuable research target due to long-term isolation. The migration of Aoluguya reindeer (AgD) is limited by mountains and traffic, which hinders genetic exchange. In recent years, as a result of this geographical isolation, habitat degradation, long-term inbreeding, population degradation, and weakened disease resistance the number of population has shown a dramatic decline (Ju et al., 2019). It is recorded that the number of reindeer is always around 1,000 and the development is not optimistic (Zhai et al., 2017). The loss of reindeer will has severe negative consequences for the environment and the indigenous cultures of the North, such as the Ewenki.

Because of the limited analyses of the genomic characteristics and genetic diversity of AgD, the genetic basis of these traits remains unclear. This also leads to a lack of relevant measures to preserve this population. So the sequencing of the AgD will be a valuable resource for researchers focused on its conservation and those interested in further reindeer genomic studies. Taking this into account, here, we report and release whole-genome sequencing data from 10 individuals with AgD to be available.

### Sample collection, DNA extraction, and sequencing

The Genhe Hongrun Green Breeding Professional Cooperative has the license to farm AgD in the Greater Khingan Mountain region of Inner Mongolia. About 2 mL blood samples of 10 unrelated adult individuals (10 males) caughted by the experienced staff were collected from the veins of tail, and all animals remained healthy after blood collection. The EasyPure Blood Genomic DNA Kit (TransGen Biotech) was used to extract genomic DNA from the blood samples. The libraries were constructed for samples which concentration of genomic DNA >0.5 μg. Finally, the qualified libraries were sequenced by a 2 × 150 bp paired-end model with DNBSEQ-T7 at Novogene Bioinformatics Institute (Beijing, China).

### Data processing and variant calling

The FASTP v0.23.2 (Chen et al., 2018) software was used to remove the adapter and low-quality sequences in raw data. Then, the clean data were mapped to the reindeer genome ULRtarCaribou_2v2 (https://www.ncbi.nlm.nih.gov/datasets/genome/GCA_019903745.2/) using BWA-MEM v0.7.17 (Li and Durbin, 2009) with default options. The Single Nucleotide Polymorphisms (SNPs) were detected by means of the Genome Analysis Toolkit (GATK v4.1.4) variant calling pipeline (McKenna et al., 2010). To reduce the false positive, only the calling quality greater than 20 was considered for these to be called. The PLINK v1.9 (Purcell et al., 2007) was used to remove the SNPs with missing rate >0.10, and minor allele frequency (MAF) < 0.15 which ensure at least 3 alleles were found in the sample set. Finally, the autosomal SNPs with only two alleles were retained.

### Data description

The FASTQ sequence data are available in NCBI Short Read Archive, which bioproject accession number is PRJNA983536. A total of 467.23 Gb clean data was retained. All individuals have been deeply sequenced with about 34.9–54.1 Gb data gained. The average mapping rate was ∼99.25% and the average depth was ∼16.8 × for each sample (Supplementary Table S1). After the SNPs detection and filtering, 8,151,569 high-quality autosomal bi-allelic SNPs (MAF ≥0.15) were identified. In these SNPs, a total of 5,487,298 (67.32%) transitions (Ts) and 2,664,271 (32.68%) transversions (Tv) were observed. The frequency of SNPs that occurred on each chromosome was different, and the average density of variants was 4.09 SNPs/Kb (Supplementary Figure S1A). The high-quality SNPs were used to measure the Linkage disequilibrium (*r* ^2^) using PopLDdecay (Zhang et al., 2019). The LD (linkage disequilibrium) decay pattern for distances up to 300 kb is shown in Figure 1B. LD value rapidly decreased with the increased distance between pair-wise SNPs, and its value (*r* ^2^ = 0.33) was about 6,500 bp in the AgD. LD levels at different distances were shown in Supplementary Table S2.

In order to get an insight into the genetic differences between AgD and other populations abroad, here we collected the resequencing data of other three populations including Fennoscandian domestic reindeer (FeD), Fennoscandian wild reindeer (FeW), and Russian wild reindeer (RuW) for comparison (Weldenegodguad et al., 2020) (Supplementary Table S3). The Principal Component Analysis (PCA) has been performed (Figures 1C, D) by Genome-wide Complex Trait Analysis (GCTA v1.92.3) (Yang et al., 2011). The plot of PC1-PC2 and PC1-PC3 all showed a clear genetic differentiation between AgD and other three populations. Moreover, the PCA pattern suggested a broader genetic differentiation within the FeW and RuW populations than in AgD.

To better understand the genetic relationship between AgD and other three populations, an unrooted maximum likelihood (ML) phylogenetic tree was constructed using RAxML (Stamatakis, 2014) software with 100 bootstraps (Figure 1E). After visualization using iTOL v6 (https://itol.embl.de/), we found AgD has a closer genetic relationship with RuW. The neighbor-joining (NJ) tree using the genetic distance estimated by PLINK v1.9 (Purcell et al., 2007) also supported the same genetic relationship (Supplementary Figure S1B). In addition, the genetic diversity indices including nucleotide diversity (*pi*), observed heterozygosity (*H*_*O*_), and expected heterozygosity (*H*_*E*_) were estimated. PLINK v1.9 (Purcell et al., 2007) with the command “--hardy” was used to perform *H*_*O*_ and *H*_*E*_, and VCFtools v0.1.16 (Danecek et al., 2011) was used to calculate *pi*. The average *H*_*O*_ and *H*_*E*_ in AgD were higher than in other three populations, and the average *pi* value also indicated a similar trend which noted that AgD has a higher genetic diversity (Table 1). This assumption can also be reflected in Supplementary Figure S1C that AgD has the lowest LD decay pattern in four populations. Finally, the method *F*_*GRM*_ was used to calculate the inbreeding coefficient of each population (Table 1). *F*_*GRM*_ was performed using the GCTA v1.92.3 (Yang et al., 2011) software with the option “--ibc”. The results showed that this AgD population has a low inbreeding level.

**TABLE 1:** Genetic diversity indices and inbreeding coefficient of four populations.

Population	*H*_*O*_	*H*_*E*_	*pi*	*F*_*GRM*_
AgD	0.285975	0.266273	0.001829	0.109599
FeD	0.247649	0.237862	0.001756	0.171377
FeW	0.228171	0.243031	0.001789	0.232228
RuW	0.254742	0.251274	0.001856	0.174014

In summary, this study provides whole-genome resequencing data of ten AgD in China, which provides a reference of data and theory for the future conservation of the valuable genetic resource AgD and the research on species of the genus *Rangifer*.

## Data Availability

The original contributions presented in the study are publicly available. This data can be found here: https://www.ncbi.nlm.nih.gov/bioproject accession number: PRJNA983536.

## References

[B1] Baskin L. M. (2000). Reindeer husbandry/hunting in Russia in the past, present and future. Polar Res. 19 (1), 23–29. 10.1111/j.1751-8369.2000.tb00324.x

[B2] Chen S. F. Zhou Y. Q. Chen Y. R. Gu J. (2018). fastp: an ultra-fast all-in-one FASTQ preprocessor. Bioinformatics 34 (17), 884–890. 10.1093/bioinformatics/bty560 30423086 PMC6129281

[B3] Danecek P. Auton A. Abecasis G. Albers C. A. Banks E. DePristo M. A. (2011). The variant call format and VCFtools. Bioinformatics 27 (15), 2156–2158. 10.1093/bioinformatics/btr330 21653522 PMC3137218

[B4] Ju Y. Liu H. M. Rong M. Zhang R. R. Dong Y. M. Zhou Y. N. (2019). Genetic diversity and population genetic structure of the only population of Aoluguya Reindeer (*Rangifer tarandus*) in China. Mitochondrial DNA Part A 30 (1), 24–29. 10.1080/24701394.2018.1448081 29658380

[B5] Kofinas G. Osherenko G. Klein D. Forbes B. (2000). Research planning in the face of change: The human role in reindeer/caribou systems. Polar Res. 19 (1), 3–21. 10.1111/j.1751-8369.2000.tb00323.x

[B6] Kvie K. S. Heggenes J. Anderson D. G. Kholodova M. V. Sipko T. Mizin I. (2016). Colonizing the high arctic: Mitochondrial DNA reveals common origin of eurasian archipelagic reindeer (*Rangifer tarandus*). Plos One 11 (11), e0165237. 10.1371/journal.pone.0165237 27880778 PMC5120779

[B7] Li H. Durbin R. (2009). Fast and accurate short read alignment with Burrows-Wheeler transform. Bioinformatics 25 (14), 1754–1760. 10.1093/bioinformatics/btp324 19451168 PMC2705234

[B8] McKenna A. Hanna M. Banks E. Sivachenko A. Cibulskis K. Kernytsky A. (2010). The genome analysis Toolkit: A MapReduce framework for analyzing next-generation DNA sequencing data. Genome Res. 20 (9), 1297–1303. 10.1101/gr.107524.110 20644199 PMC2928508

[B9] Purcell S. Neale B. Todd-Brown K. Thomas L. Ferreira M. A. R. Bender D. (2007). Plink: A tool set for whole-genome association and population-based linkage analyses. Am. J. Hum. Genet. 81 (3), 559–575. 10.1086/519795 17701901 PMC1950838

[B10] Stamatakis A. (2014). RAxML version 8: A tool for phylogenetic analysis and post-analysis of large phylogenies. Bioinformatics 30 (9), 1312–1313. 10.1093/bioinformatics/btu033 24451623 PMC3998144

[B11] Weldenegodguad M. Pokharel K. Ming Y. Honkatukia M. Peippo J. Reilas T. (2020). Genome sequence and comparative analysis of reindeer (*Rangifer tarandus*) in northern Eurasia. Sci. Rep. 10 (1), 8980. 10.1038/s41598-020-65487-y 32488117 PMC7265531

[B12] Yang J. A. Lee S. H. Goddard M. E. Visscher P. M. (2011). Gcta: A tool for genome-wide Complex trait analysis. Am. J. Hum. Genet. 88 (1), 76–82. 10.1016/j.ajhg.2010.11.011 21167468 PMC3014363

[B13] Zhai J. C. Liu W. S. Yin Y. J. Xia Y. L. Li H. P. (2017). Analysis on genetic diversity of reindeer (*Rangifer tarandus*) in the greater khingan mountains using microsatellite markers. Zool. Stud. 56, e11. 10.6620/ZS.2017.56-11 31966210 PMC6517736

[B14] Zhang C. Dong S. S. Xu J. Y. He W. M. Yang T. L. (2019). PopLDdecay: A fast and effective tool for linkage disequilibrium decay analysis based on variant call format files. Bioinformatics 35 (10), 1786–1788. 10.1093/bioinformatics/bty875 30321304

