# Supplementary figures and images for: Whole genome sequencing of Aoluguya reindeer (Rangifer tarandus) in China
Source: Front Genet. 2023 Aug 2;14:1243795. doi: 10.3389/fgene.2023.1243795 (PMC10433215; doi:10.3389/fgene.2023.1243795)

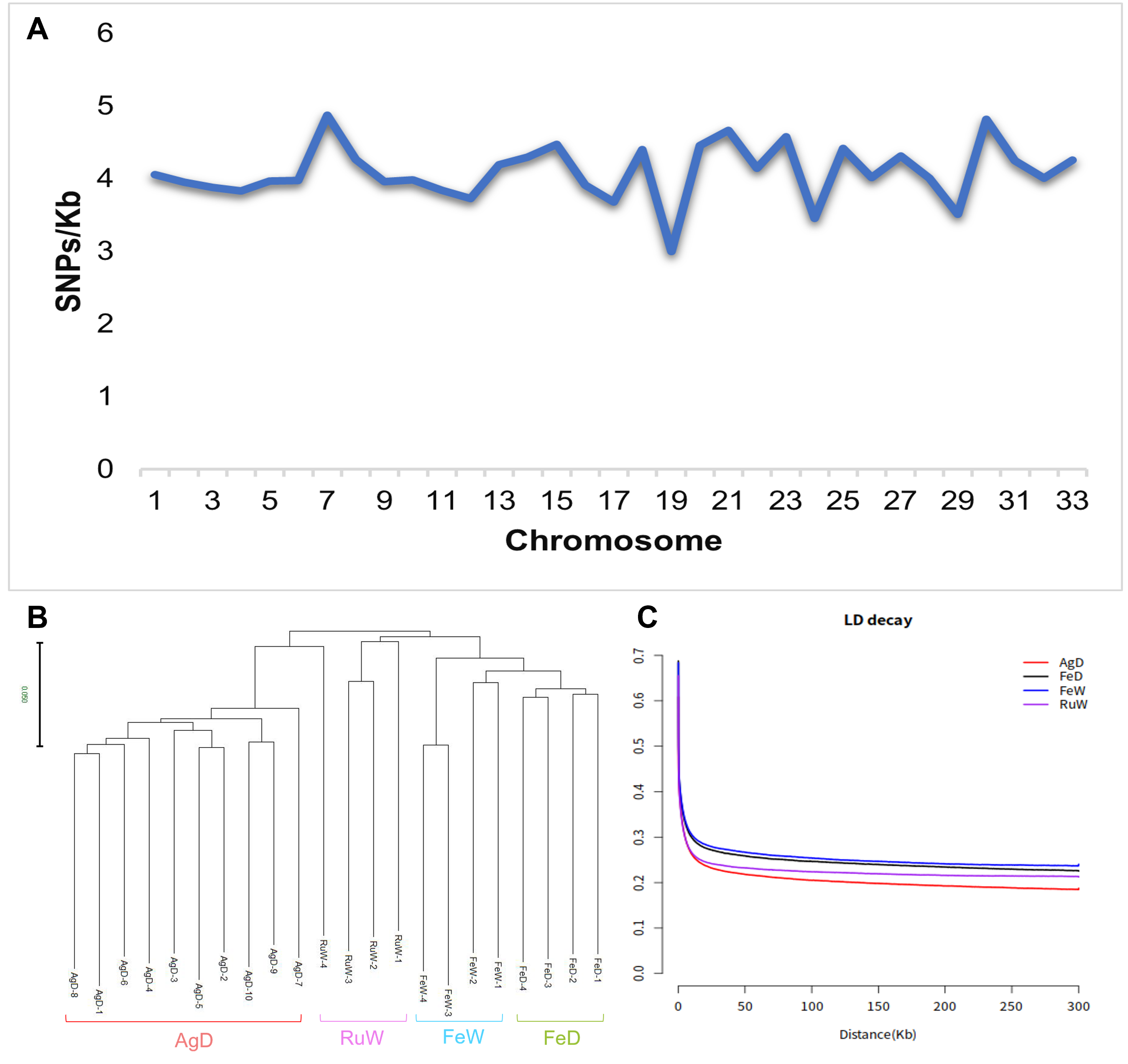

Supplement: Supplementary file 4 [file Image1.PNG]
